# First person – Yunlong Li

**DOI:** 10.1242/bio.041046

**Published:** 2019-01-15

## Abstract

First Person is a series of interviews with the first authors of a selection of papers published in Biology Open, helping early-career researchers promote themselves alongside their papers. Yunlong Li is first author on ‘The ATPase TER94 regulates Notch signalling during Drosophila wing development’, published in BiO. Yunlong is a graduate student in the lab of Junzheng, Zhang at China Agricultural University, Beijing, P. R. China, investigating the regulatory mechanisms of developmental signalling pathways in *Drosophila melanogaster*.


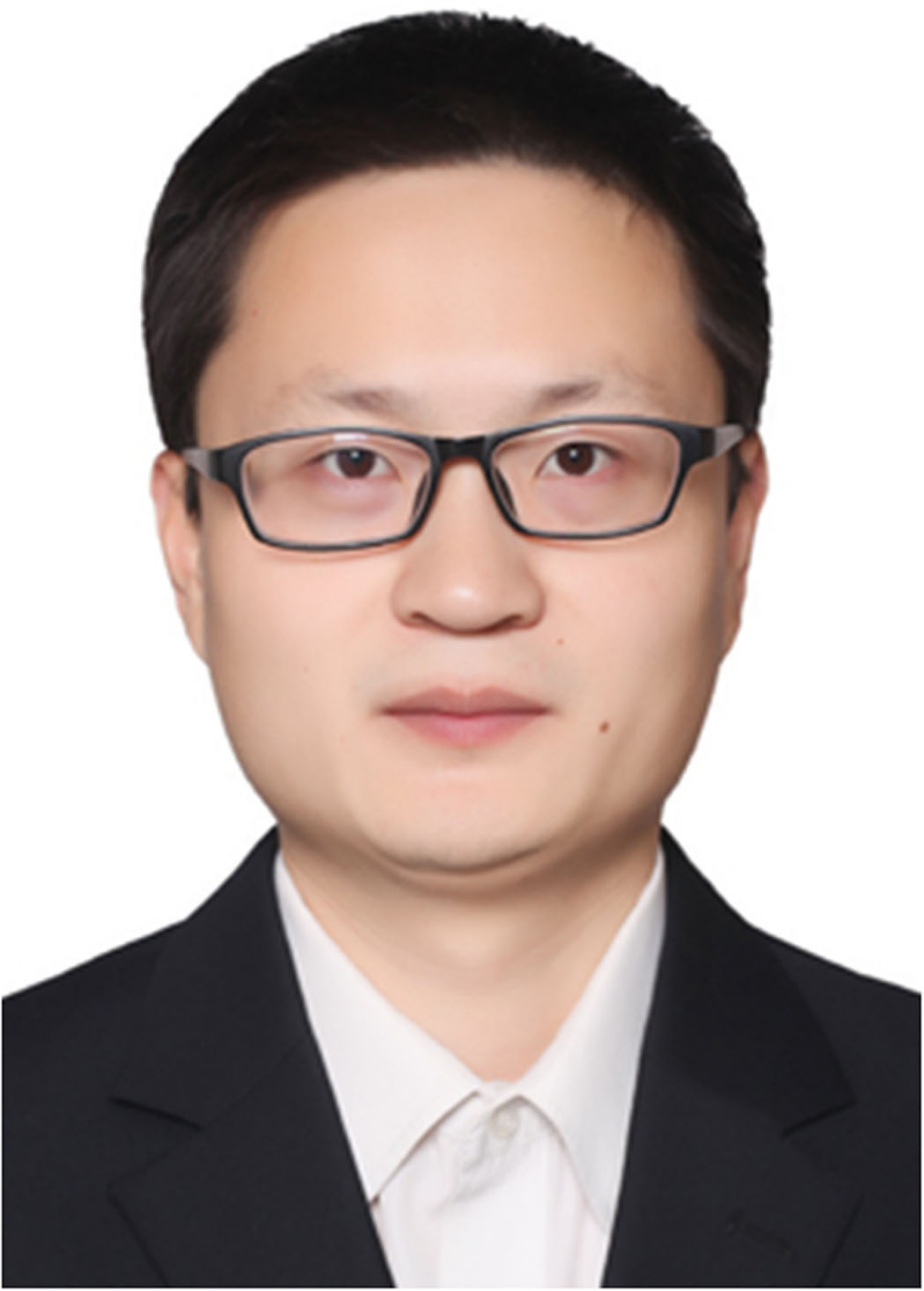


**Yunlong Li**

**What is your scientific background and the general focus of your lab?**

I hold a Bachelor's degree of Science in Agriculture. Currently, I am a graduate student in the Department of Entomology, College of Plant Protection, China Agricultural University. Mentored by Dr Junzheng Zhang, I am in my third year of the Master's degree program. Our lab focuses on understanding the molecular mechanism of insect organ development.

**Figure:**
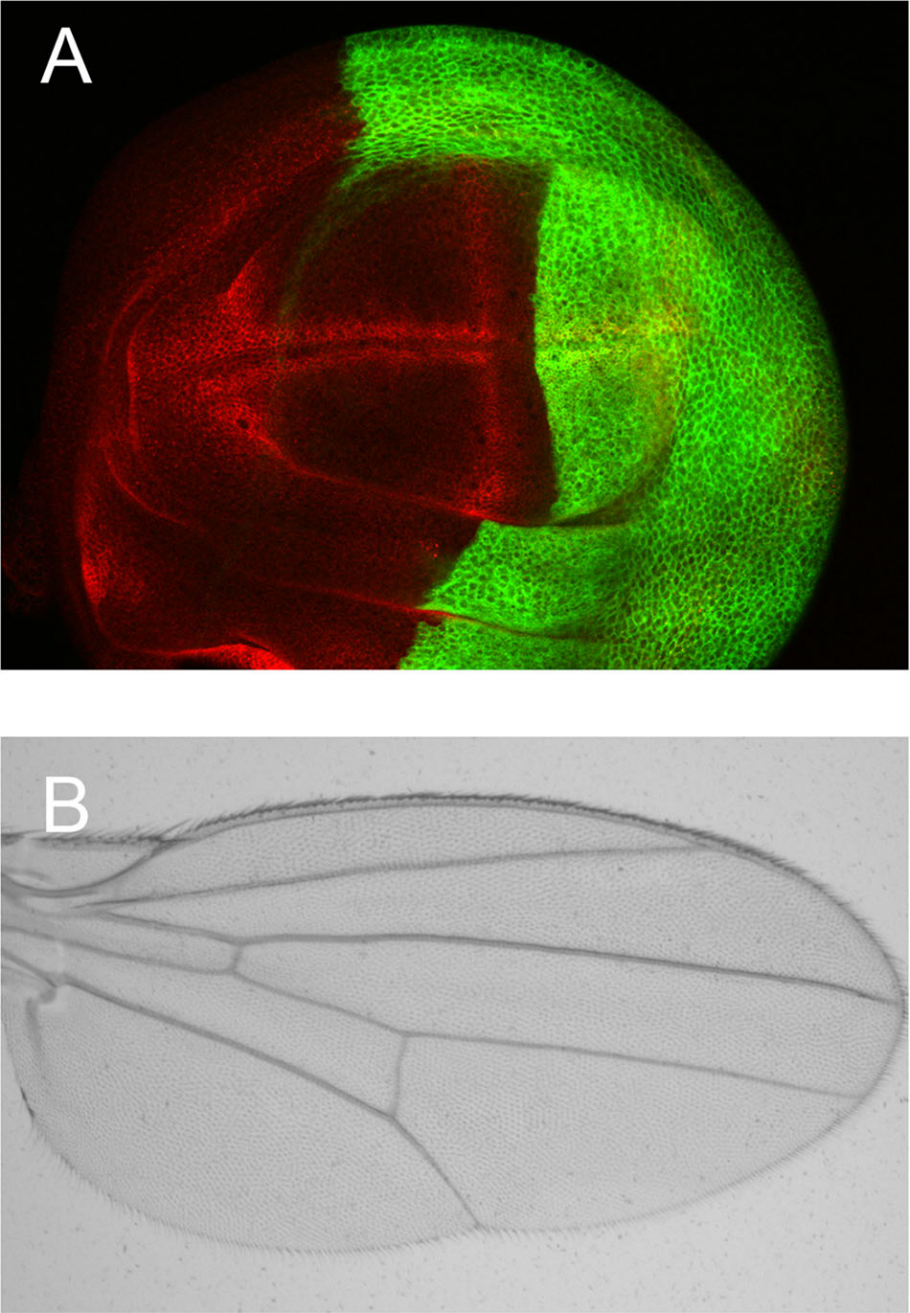
***Drosophila* wing.**

**How would you explain the main findings of your paper to non-scientific family and friends?**

All insects have wings and the formation of wings is controlled by a group of genes. We found one such gene that is required for wing formation in fruit flies, and possibly for many other insects.

**What are the potential implications of these results for your field of research?**

We found that the TER94 ATPase was as a novel regulator of the Notch signalling pathway in *Drosophila*, thus it reveals a novel role of TER94 in development.

**What has surprised you the most while conducting your research?**

The fact that *Drosophila* TER94 is able to regulate the activity of multiple signalling pathways, potentially through distinct co-factors.

**What, in your opinion, are some of the greatest achievements in your field and how has this influenced your research?**

For me, the greatest achievements in developmental biology includes the discovery of signalling pathway crosstalk and the establishment of FLP-FRT system in *Drosophila*. We are able to dissect the function of vital genes in organ development by the FLP-FRT system. And we are beginning to appreciate that no signalling pathway plays a solo role during development, as more and more factors are found to regulate multiple pathways simultaneously.

**What changes do you think could improve the professional lives of early-career scientists?**

Three things: (1) the government and relevant departments should set up a number of foundations to provide funds for scientific research and teaching activities for young scientific researchers, (2) the schools should free early-career scientists from pointless meetings to focus on science, and (3) independent critical thinking needs to be cultivated in young scientists.

**What's next for you?**

Hopefully, I can pursue my doctorate degree in one of the world's top universities.
